# Polygenic risk for epigenetic aging and adverse life experiences interact to predict growth in adolescent depression in a racially/ethnically diverse sample

**DOI:** 10.3389/fpsyt.2024.1499395

**Published:** 2024-12-20

**Authors:** Kit K. Elam, Jinni Su, Weisiyu Abraham Qin, Kathryn Lemery-Chalfant

**Affiliations:** ^1^ Department of Applied Health Science, School of Public Health, Indiana University, Bloomington, IN, United States; ^2^ Psychology Department, Arizona State University, Tempe, AZ, United States

**Keywords:** polygenic, epigenetics, depression, adverse life events, parent acceptance, early adolescence

## Abstract

**Introduction:**

Research has yet to examine the interplay between indices of environmental risk and resilience processes and genetic predisposition for epigenetic aging in predicting early adolescent depressive symptoms. In the current study we examine whether adverse life events and parental acceptance moderate polygenic predisposition for GrimAge epigenetic aging in predicting trajectories of depressive symptoms across early adolescence.

**Method:**

Using data from the Adolescent Brain Development Study (ABCD, N = 11,875), we created polygenic scores for GrimAge, and examined whether exposure to adverse life events and parental acceptance moderated the relation between genetic risk and depressive symptom trajectories from age 10/11 to 12/13 using growth mixture modelling. We examined models separately in European American (EA), African American (AA), and Latinx (LX) subgroups of ABCD.

**Results:**

In the EA and AA subgroups, adverse life events moderated polygenic scores for GrimAge such that there was increased likelihood of membership in a higher vs. lower depression trajectory.

**Discussion:**

We extend literature by identifying genetic contributions to epigenetic aging as a depression diathesis in adolescence. Findings also highlight the detrimental role of adverse life events in exacerbating genetic risk for the development of depression in adolescence.

## 1 Introduction

### 1.1 Epigenetic aging

Developmental science is replete with examples of environmental stress-induced changes to the epigenome that then influence protein expression and downstream physiology, emotion, cognition, and behavior, including mental health symptoms and disorders. Seldom studied are the genetic influences on epigenetic variation, such as histone acetylation and methylation. Some alleles, for example, are more likely to be methylated than others, necessitating the study of genetic influences on epigenetic variation. Epigenetic clocks capture combinations of DNA methylation patterns (1). Biological age indexed by epigenetic clocks are more reliable measures of aging than chronological age (2), and importantly, variation in these clocks predicts mental health problems such as depression (3, 4). Accumulating evidence indicates that epigenetic clocks are heritable (5, 6). The second-generation epigenetic clock GrimAge is currently the best predictor of health span and lifespan (7). A recent Genome Wide Association Study (GWAS) (6) generated summary effect sizes for SNP prediction of GrimAge, providing the statistics needed for forming a polygenic risk score representing genomic influences on GrimAge. Polygenic scores aggregate variation across thousands of genetic variants across the genome, providing a cumulative measure of genetic predisposition (8). In the current study, we present a novel examination of whether polygenic predisposition for GrimAge epigenetic aging is moderated by adverse life events, as a risk factor, and parent acceptance, as a resilience factor, in predicting early adolescent depression.

### 1.2 Theoretical foundation

Developmental psychopathology theory emphasizes the cumulative salience of multiple levels of risk and resilience within the social environment over the course of psychopathology (9, 10). Separate from this, the diathesis-stress theory of depression proposes that preexisting risk factors, such as genetic predisposition, can be exacerbated by risky environments, increasing likelihood of depression (11, 12). Conversely, within a resilience framework, positive environmental influences may buffer genetic predisposition for depression (13). As an extension and integration of these theories, the gene-environment cascade theoretical framework posits that that the interplay between genetic predisposition and the environment can have cascading effects that alter trajectories of mental health over time (14). Leveraging this framework, we expand traditional main effect genetic association models to include risk and resilience processes and developmental outcomes. Furthermore, we account for the known heterogeneity in depression by allowing for developmental subgroups of depression using growth mixture modeling.

### 1.3 Adverse life events

Adverse life events refers to experiences or exposure to traumatic or stressful events. A robust literature including a recent meta-analysis indicates that adverse life events are associated with greater incidence of major depressive disorder before the age of 18 (15). The associations between adverse life events and increases in depression and depressive symptoms in childhood and adolescence have also been corroborated in large international and national samples (16, 17). Thus, adverse life events represent a compelling index of environmental risk during childhood. Moreover, there is ample evidence that risk for depression results from the interplay between adverse life events and genetic and epigenetic predispositions.

A recent review concluded that the interplay between genetic predisposition and adverse life events in childhood represents a classic example of gene-environment interaction (GxE) in mental disorder (18). This is supported by an umbrella review indicating that childhood risk for depression is consistent with a diathesis-stress framework resulting, in part, from adverse environmental exposure and genetic and/or epigenetic predispositions (19). Moreover, this review and its cited literature notes that variability in DNA methylation results from both genetic and environmental variation (20). This aligns with another review of biological factors in depression indicating that one route by which adverse life events in childhood contribute to depression is via the interplay between genetic and epigenetic systems (21). Thus, there is theoretical and research evidence indicating that genetic predisposition for epigenetic variation may be moderated by adverse life events in predicting depressive symptoms in early adolescence.

### 1.4 Parent acceptance

Parent acceptance encompasses warm, involved, and supportive behaviors a parent expresses towards their child. Four meta-analysis support associations between greater parental warmth and lower childhood/adolescent depression (22–25). Compared to GxE research on adverse life events, less research has examined parent acceptance as moderating genetic predisposition in predicting depression. One study found an interaction between polygenic predisposition for genetic sensitivity and parental warmth associated with depressive symptoms, but the polygenic index was based on only a few candidate genes (26). Using a twin model, parent nurturance moderated heritability for psychological resilience (27). More commonly, GxE studies have examined single candidate genes, negative aspects of parenting, and externalizing outcomes (28). This prompts the need for greater examination of positive parenting as a resilience factor operating as a buffer between genetic risk and pathways to early adolescent depression.

### 1.5 Current study

The current study examined whether genetic predisposition for GrimAge epigenetic aging was moderated by adverse life events and parent acceptance in predicting depressive symptom trajectories across early adolescence. Due to known heterogeneity in depression development, we used a person-centered approach to uncover depressive symptom classes across early adolescence separately within EA, AA, and LX subgroups. Person-centered approaches identify distinct patterns of behavior for subgroups of individuals whereas variable-centered approaches collapse variance in a behavior across individuals which can miss heterogeneity in the group (29). Growth Mixture Modelling (GMM) is one person-centered approach that identifies subgroups based on patterns of individual-level trajectories on a specific trait or behavior (30). We examined trajectories and genetic effects separately in EA, AA, and LX subgroups because there can be underlying variation in genetic ancestry across race and ethnicity (e.g., allele frequency, linkage disequilibrium patterns) which necessitate examining effects separately within each racial/ethnic group (31).

We hypothesized that genetic predisposition for GrimAge would be associated with greater likelihood of membership in trajectories with higher depressive symptoms. We hypothesized that adverse life events would moderate the effect of polygenic predisposition for GrimAge such that the association between genetic risk and membership in higher vs. lower depressive symptom trajectories would be stronger among youth experiencing more adverse life events. Conversely, we hypothesized that the association between genetic risk and membership in higher depressive symptom vs. lower trajectories would be weaker among youth who report higher levels of parental acceptance.

## 2 Method

### 2.1 Participants

The Adolescent Brain Cognitive Development (ABCD; *N* = 11,875) Study is a large longitudinal study of youth in the United States with annual assessments on behavioral, social, and neurocognitive functioning starting at age 9/10 with four waves of data available to age 12/13. Youth in ABCD are 47.8% female and racially/ethnically diverse (52.1% non-Hispanic White, 15.0% non-Hispanic Black, 20.3% Hispanic/Latinx, 2.1% Asian, and 10.5% other [e.g., multiracial]). At age 9/10, median combined family income was $75k to $100k, with approximately 20% of the sample reporting earning $35k or less. The current study included data from the age 10/11, 11/12, and 12/13 assessments. Youth were included if they had genetic, depressive symptom, and environmental data and were White/European American (EA, *n* = 6,043; 47% female), Black/African American (AA, *n* = 1,640; 50% female), or Hispanic/Latinx (LX, *n* = 2,283; 48% female).

### 2.2 Procedures

Youth in ABCD were primarily recruited using a probability sampling of schools located within 21 national study sites (32). Parents provided written informed consent for their own and their child’s participation and youth provided assent. Baseline data collection for age 9/10 began in September, 2016, with annual follow ups. Youth in ABCD are assessed in an array of domains encompassing psychosocial and family functioning, physical health, contextual and cultural environment, brain imaging, and whole genome genotyping. Data are released through the NIMH Data Archive. Data used in the present study came from ABCD data release 5.0.

Saliva samples were collected from youth at the age 9/10 assessment (33), which were genotyped by the Rutgers University Cell and DNA Repository (RUCDR). DNA from saliva samples was genotyped on the Smokescreen Genotyping Array (34). RUCDR performed DNA quality controls based on calling signals and variant call rates, and the quality-controlled genotyping ABCD data contains 11,099 unique individuals with 516,598 genetic variants. Imputation was performed via the TOPMed imputation server using mixed ancestry and Engle v2.4 phasing. Single nucleotide polymorphisms (SNPs) with a genotyping rate < 0.95 or that violated Hardy–Weinberg equilibrium (p < 10^-6^) or with minor allele frequency < 0.01 were excluded from analysis.

### 2.3 Measures

#### 2.3.1 Polygenic scores for GrimAge

We created the PGS based on summary statistics from McCartney et al. (6), the largest published trans-ancestral genome-wide association study (GWAS) on DNA methylation biomarkers of aging. In McCartney et al., GWAS were performed on multiple indices of DNA methylation in approximately 40,000 individuals separately for European American and Black/African American samples (European American *N* = 34,710; Black/African American *N* = 6,195). We leveraged summary statistics to create a PGS characterizing genetic predisposition for GrimAge (Grim-PGS) in ABCD after filtering and matching discovery, target, and reference samples. We formed Grim-PGS using the PRS-CSx method, which uses a Bayesian regression and continuous shrinkage method (35, 36). Using GWAS summary statistics, PRS-CSx estimates posterior effect sizes for SNPs which are inferred under coupled continuous shrinkage priors across multiple populations, yielding more accurate effect size estimation. Grim-PGS scores were calculated using joint modelling across EA and AA GWAS summary statistics via coupled shrinkage priors (36). Final Grim-PGS were based on posterior PRS-CSx weights and created using the *score* procedure in PLINK 1.9 (37). Given that no GWAS on DNA methylation exist in Latinx samples, we used Grim-PGS meta-analyzed across EA and AA GWAS generated by PRS-CSx, which have shown enhanced portability across populations (36). An overview on the underlying principes of polygenic score creation can be found in Kachuri et al. (2024) (38).

#### 2.3.2 Population stratification and genetic admixture

To account for potential population stratification, within each ancestry, the first 10 genetic ancestry principal components were extracted based on ancestry informative markers, which were residualized from the Grim-PGS for each ancestral group. Final Grim-PGS were standardized for ease of interpretation.

#### 2.3.3 Adverse life events (Age 10/11)

Youth reported on their significant life events using the Adverse Life Events scale (39–43). This scale captures youth experience of adverse life events on 25 items (e.g., “Saw crime or accident”, “Mother or father lost job”; *yes* (1) or *no* (0)). Each item was followed by youth’s perception of the event (“Was this a good or bad experience?”; *mostly good* (1) or *mostly bad* (2)). Total number of negative adverse life events were summed and youth experiencing greater than 10 events were recoded as 11 to help address sparsity of datapoints (<1%), limit the influence of extreme values, and in-line with past research on adverse experiences (44, 45).

#### 2.3.4 Parent acceptance (Age 10/11)

Youth reported on parenting using the Child Report of Behavior Inventory (46, 47). Five items from the acceptance subscale capture youth perception of their parent’s warmth, acceptance, and responsiveness (e.g., “Is able to make me feel better when I am upset”). Response options ranged from 1 (not at all) to 3 (very much). Items were coded to reflect greater acceptance and mean composited (Cronbach’s alpha = .72).

#### 2.3.5 Depressive symptoms (Age 10/11, 11/12, 12/13)

Parents reported on their child’s behavior using the Child Behavior Check List (48). The DSM-oriented depression subscale captures depressive symptoms during the past 6 months based on 13 items (e.g., “Unhappy, sad, or depressed” and “There is very little s/he enjoys”) on a three-point scale; *not true* (0) to *very true/often true* (2). Items were coded to reflect greater depressive symptoms and mean composited within study wave (Cronbach’s alphas ranged from .74 to .79).

#### 2.3.6 Covariates

Extant research indicates that both depression and epigenetic aging can vary by sex, pubertal status, and income (49–51). Therefore, we controlled for sex recoded to binary for analyses (1 = male, 2 = female), income (1 = less than $5000 to 10 = $200,000 and greater), and pubertal status (1 = prepuberty to 5 = postpuberty) and examined Grim-PGS by covariate interactions (52).

### 2.4 Analytic approach

There are potential differences in polygenic score functioning across racial and ethnic groups based on genetic ancestry (31). Therefore, we conducted all analyses separately by racial/ethnic subgroup. Using Growth Mixture Modelling (GMM), we examined trajectories of depressive symptoms across ages 10-11, 11-12, and 12-13 separately for EA, AA, and LX subgroups in Mplus v.8.8 including intercept, slope, and quadratic terms. We did not include wave 9/10 depressive symptoms in GMM as youth report of adverse life events was not available until age 10/11. We examined iterative GMM solutions starting with 1 class and increasing to 5 classes. We examined model fit based on Akaike Information Criterion (AIC), Bayesian Information Criterion (BIC), adjusted BIC (aBIC), Vuong-Lo-Mendell-Rubin Likelihood Ratio Test (VLMR), Bootstrap Likelihood Ratio Test (BLRT), entropy, class sample proportions, and theoretical interpretability (53). Full information maximum likelihood was used to handle missing data based upon the missing at random (MAR) assumption.

Once we identified the optimal class solution, we examined associations between our predictors and covariates with depression trajectories separately within the EA, AA, and LX groups, using the R3step method (54). The R3step method estimates latent classes based on indicators (e.g., depression), creates a most likely latent class variable based on the posterior distribution, and regresses the predictor(s) on class membership. The R3step method tests for likelihood of trajectory membership across pairwise class comparisons which we examined using the lowest depression trajectory as the reference group.

Within the optimal class solution, we tested two models within each subgroup. The first model examined associations between the Grim-PGS, adverse life events, the Grim-PGS by adverse life events interaction term, and covariates with depression trajectories. The second model examined associations between the Grim-PGS, parent acceptance, the Grim-PGS by parent acceptance interaction term, and covariates with depression trajectories. Based on methodological recommendations, within all models we examined for Grim-PGS by covariate interactions, and non-significant interactions were trimmed from final models (52).

## 3 Results

### 3.1 Descriptive statistics

Descriptive statistics and correlations can be found in **Table 1**. There were relatively low levels of adverse life events and relatively high levels of parent acceptance across EA, AA, and LX subgroups. Depressive symptoms were also low in all subgroups. There were significant racial/ethnic differences in youth’s experience of adverse life events, with the highest levels in the AA subgroup and lowest levels in the EA subgroup. Parental acceptance was highest in the EA subgroup and lowest in the AA subgroup. Depressive symptoms were lower in the AA subgroup compared to both the EA and LX subgroups. Within the EA subgroup the Grim-PGS was associated with lower parent acceptance and greater depression at age 10/11. Within all subgroups, adverse life events were associated with higher youth depression, and parent acceptance was associated with lower youth depression.

**Table 1:** Means, standard deviations, and bivariate correlations between study variables by racial/ethnic group.

	Grim-PGS	Significant Life Events	Parent Acceptance	Depression age 10/11	Depression age 11/12	Depression age 12/13	Pubertal Status	Age	Sex	Income
European American										
Grim-PGS	1									
Adverse life Events	-0.012	1								
Parent Acceptance	-0.027*	-0.144**	1							
Depression age 10/11	0.026*	0.143**	-0.146**	1						
Depression age 11/12	0.009	0.143**	-0.143**	0.622**	1					
Depression age 12/13	0.005	0.121**	-0.103**	0.551**	0.619**	1				
Pubertal Status	-0.007	0.023	0.007	0.012	0.045**	0.084**	1			
Age	0.010	-0.014	0.032*	0.020	0.056**	0.018	0.245**	1		
Sex	-0.008	-0.047**	0.057**	-0.065**	-0.017	0.029	0.592**	-0.018	1	
Income	-0.023	-0.204**	0.109**	-0.147**	-0.135**	-0.114**	-0.068**	0.031*	0.014	1
M (SD) or %	-.01 (1.05)	2.18 (2.03)	2.83 (.27)	1.40 (2.15)	1.51 (2.24)	1.73 (2.47)	1.92 (.94)	10.95 (.65)	47%	8.22 (1.69)
African American										
Grim-PGS	1									
Adverse life Events	0.027	1								
Parent Acceptance	-0.014	-0.081**	1							
Depression age 10/11	-0.010	0.086**	-0.090**	1						
Depression age 11/12	0.045	0.075*	-0.092**	0.594**	1					
Depression age 12/13	-0.038	0.099**	-0.134**	0.575**	0.618**	1				
Pubertal Status	-0.010	-0.057*	0.030	0.008	-0.006	-0.005	1			
Age	0.006	-0.048	-0.056*	0.020	0.034	0.009	0.221**	1		
Sex	-0.002	-0.057*	0.045	-0.034	-0.026	0.024	0.581**	-0.042	1	
Income	0.031	-0.100**	0.017	-0.059*	-0.068*	-0.014	-0.025	0.056*	0.022	1
M/SD or %	.02 (1.06)	3.27 (2.63)	2.75 (.33)	1.20 (2.13)	1.08 (1.89)	1.19 (2.08)	2.57 (.95)	10.92 (.63)	50%	4.97 (2.65)
Latinx										
Grim-PGS	1									
Adverse life Events	0.011	1								
Parent Acceptance	-0.010	-0.081**	1							
Depression age 10/11	0.025	0.086**	-0.090**	1						
Depression age 11/12	0.008	0.075*	-0.092**	0.594**	1					
Depression age 12/13	-0.030	0.099**	-0.134**	0.575**	0.618**	1				
Pubertal Status	0.009	-0.057*	0.030	0.008	-0.006	-0.005	1			
Age	-0.004	-0.048	-0.056*	0.020	0.034	0.009	0.221**	1		
Sex	-0.003	-.057*	0.045	-0.034	-0.026	0.024	0.581**	-0.042	1	
Income	-0.004	-.100**	0.017	-0.059*	-0.068*	-0.014	-0.025	0.056*	0.022	1
M/SD or %	.03 (1.02)	2.62 (2.31)	2.80 (.29)	1.48 (2.31)	1.48 (2.29)	1.68 (2.52)	2.16 (1.01)	10.87 (.65)	48%	6.15 (2.46)
Mean differences across EA, AA, LX	–	AA>LX>EA	EA>LX>AA	EA>AA; LX>AA	EA>AA; LX>AA	EA>AA; LX>AA	AA>LX>EA	EA>LX	Ns	EA>LX>AA

EA, European American; AA, African American; LX, Latinx; Ns, nonsignificant differences.

*p < .05, **p < .01.

### 3.2 Depression symptom trajectories

**Figure 1:**
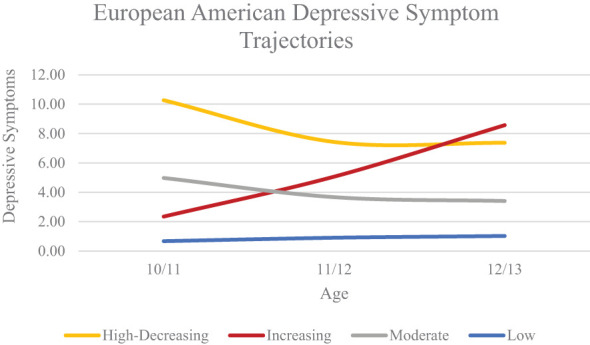
European American Depressive Symptom Trajectories Across Early Adolescence.

We conducted growth mixture modelling separately within the EA, AA, and LX subgroups, increasing the number of classes iteratively. Initial models had better fit without the quadratic term in all subgroups, so we excluded the quadratic slope from our iterative model testing. Model fit indices can be found in **Table 2**. For the EA subgroup, a four-class model was optimal based on lower AIC, BIC, and aBIC values compared to the 3-class model and significant VLMR and BLRT values compared to the 5-class model. Average levels of depressive symptoms can be found in **Figure 1**. We classified the four classes as high-decreasing (n = 138; 2.3%), increasing (n = 201; 3.3%), moderate (n = 633; 10.5%), and low (n = 5,071; 83.9%).

**Table 2:** Latent class analysis model fit indices.

AA Class	AIC	BIC	aBIC	Entropy	VLMR *p-*value	BLRT *p*-value	Smallest Class Size
1	13882.79	13926.01	13900.59	–	–	–	–
2	13062.21	13121.64	13086.69	.97	.012	.014	7%
**3**	**12567.66**	**12643.29**	**12598.81**	**.96**	**.011**	**.013**	**2%**
4	12299.05	12390.89	12336.88	.93	.29	.30	2%
5	12174.12	12282.17	12218.63	.91	.32	33	2%
EA Class	AIC	BIC	aBIC	Entropy	VLMR p-value	BLRT p-value	Smallest Class Size
1	61769.85	61823.50	61798.08	–	–	–	–
2	59334.58	59409.36	59374.40	.95	<.001	<.001	8%
3	57839.12	57933.01	57888.53	.94	.001	.001	2%
**4**	**56922.23**	**57036.25**	**56982.22**	**.92**	**.04**	**.04**	**2%**
5	56383.13	56517.26	56453.71	.92	.62	.63	1%
LX Class	AIC	BIC	aBIC	Entropy	VLMR p-value	BLRT p-value	Smallest Class Size
1	225511.56	22557.43	22532.01	–	–	–	–
**2**	**21474.81**	**21537.87**	**21502.92**	**.95**	**.04**	**.04**	**6%**
3	20898.63	20978.89	20934.41	.94	.06	.06	3%
4	20510.10	20607.56	20553.55	.92	.02	.03	2%
5	20247.50	20362.16	20298.62	.92	.42	.42	1%

EA, European American; AA, African American; LX, Latinx. Bolded class indicates optimal solution.

**Figure 2:**
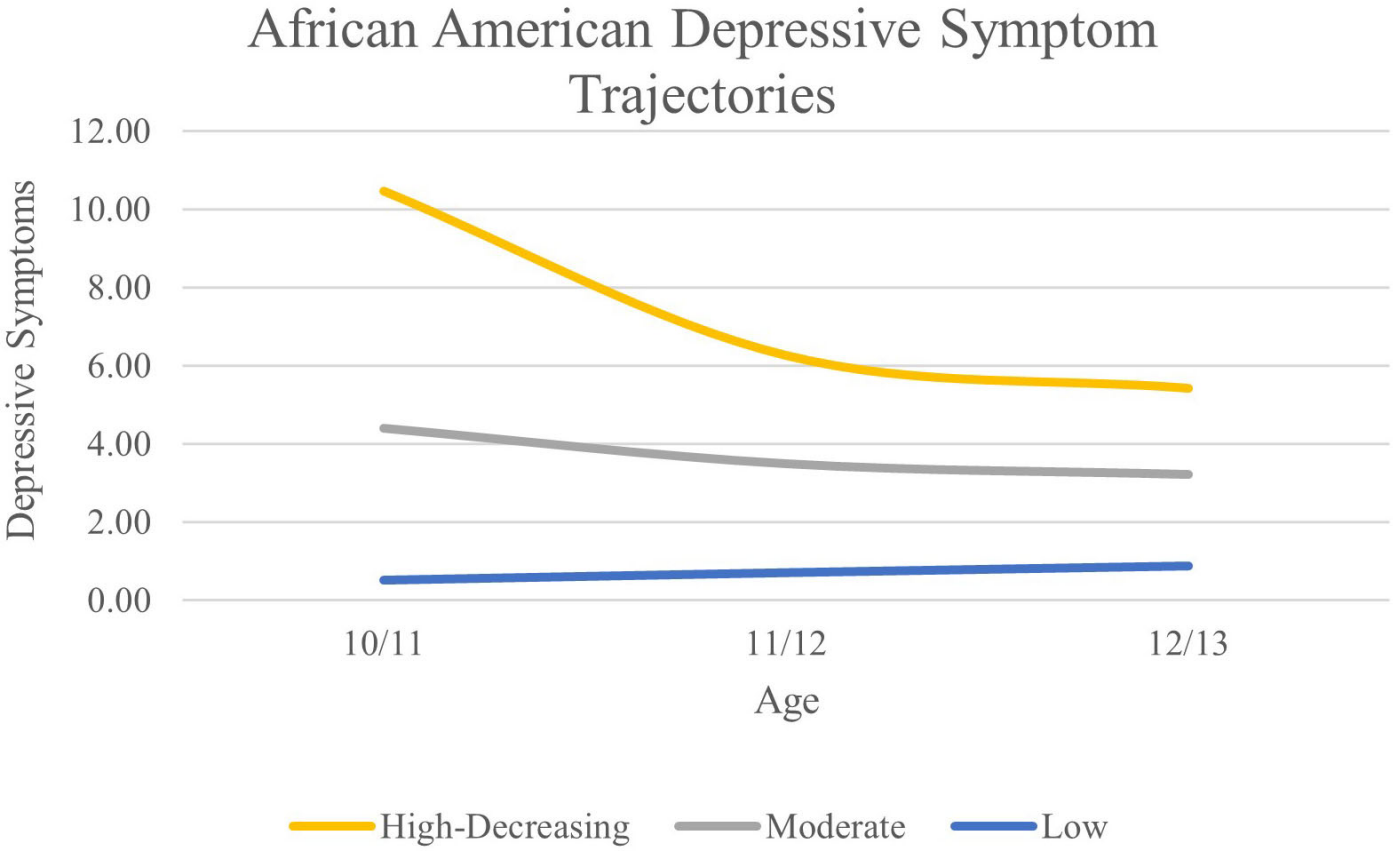
African American Depressive Symptom Trajectories Across Early Adolescence.

For the AA subgroup, a three-class solution was optimal based on lower AIC, BIC, and aBIC values compared to the 2-class solution and significant VLMR and BLRT values compared to the 4-class solution. Average levels of depressive symptoms can be found in **Figure 2**. We classified the three classes as high-decreasing (n = 40; 2.4%), moderate (n = 183; 11.2%), and low (n = 1,417; 86.4%).

**Figure 3:**
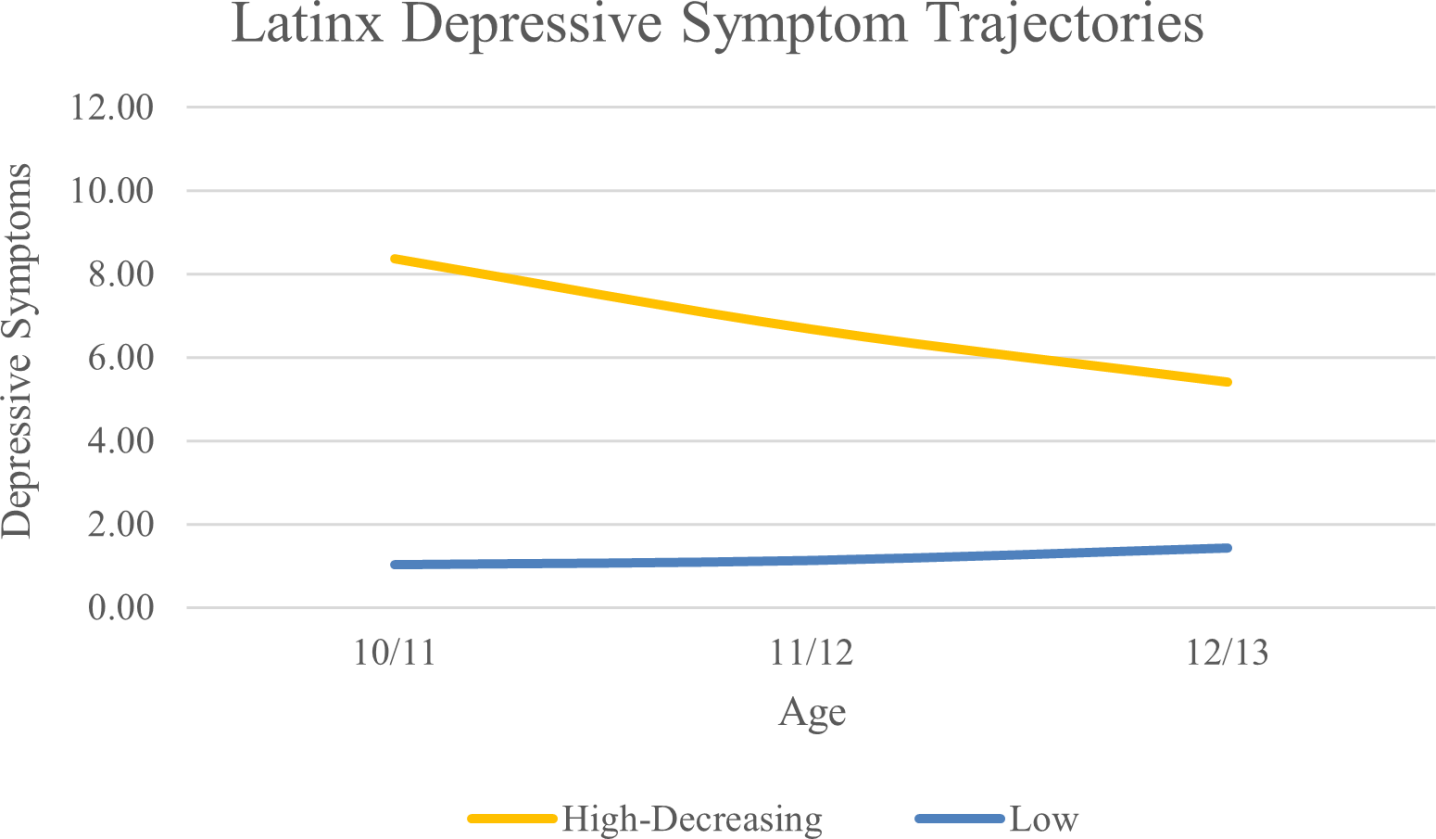
Latinx Depressive Symptom Trajectories Across Early Adolescence.

For the LX subgroup, a two-class solution was optimal based on lower AIC, BIC, and aBIC values compared to the 1-class solution and significant VLMR and BLRT values compared to the 3-class solution. Average levels of depressive symptoms can be found in **Figure 3**. We classified the two classes as high-decreasing (n = 140; 6.1%) and low (n = 2143; 93.9%).

### 3.3 Associations with depressive symptom trajectories

Associations between Grim-PGS, adverse life events, and parental acceptance and depressive symptom trajectories can be found in **Tables 3**–**5**. Within the EA subgroup (see **Table 3**), adverse life events were associated with greater likelihood of membership in the high-decreasing, increasing, and moderate depression trajectories compared to the low depression trajectory. The Grim-PGS by adverse life events interaction was associated with greater likelihood of being in the moderate vs. low trajectory. Follow up analyses indicated that the association between Grim-PRS and membership in the moderate vs. low trajectory was stronger among youth who reported experiencing greater adverse life events (+.5SD; OR = 1.35, 95% CI [1.05, 1.74]) than among youth who experienced fewer adverse life events (-.5SD, OR = .90, 95% CI [.48, 1.71]). Parental acceptance was associated with lower likelihood of following the high, increasing, and moderate trajectories compared to the low trajectory. There were no significant interaction effects between Grim-PGS and parental acceptance in predicting trajectories of depressive symptoms.

**Table 3:** Associations between Grim-PGS, adverse life events, parental acceptance, and depressive symptom trajectories among European American youth.

Comparison	Variable	OR	95% CI	OR	95% CI
High-Decreasing vs. Low	Grim-PGS	1.069	0.912, 1.253	1.054	0.858, 1.295
	Adverse life Events	**1.521**	**1.292, 1.791**	**-**	**-**
	Grim-PGS by ALE	0.937	0.818, 1.072	–	–
	Parent Acceptance	–	–	**0.700**	**0.602, 0.814**
	Grim-PGS by Acceptance	–	–	1.086	0.895, 1.319
	Sex	1.302	0.844, 2.008	**0.475**	**0.285, 0.792**
	Income	0.961	0.868, 1.064	**0.732**	**0.676, 0.793**
	Pubertal Status	**1.581**	**1.259, 1.986**	**1.454**	**1.135, 1.864**
Increasing vs. Low	Grim-PGS	1.037	0.828, 1.299	1.045	0.895, 1.22
	Adverse life Events	**1.554**	**1.297, 1.863**	**-**	**-**
	Grim-PGS by ALE	1.144	0.963, 1.358	**-**	**-**
	Parent Acceptance	–	–	**0.763**	**0.645, 0.903**
	Grim-PGS by Acceptance	–	–	1.133	0.982, 1.307
	Sex	**0.471**	**0.282, 0.786**	1.285	0.837, 1.973
	Income	**0.758**	**0.694, 0.827**	0.926	0.838, 1.024
	Pubertal Status	**1.415**	**1.095, 1.829**	**1.586**	**1.27, 1.98**
Moderate vs. Low	Grim-PGS	1.044	0.949, 1.147	1.031	0.938, 1.132
	Adverse Life Events	**1.255**	**1.132, 1.393**	**-**	**-**
	Grim-PGS by ALE	**1.114**	**1.004, 1.237**	**-**	**-**
	Parent Acceptance	–	–	**0.722**	**0.657, 0.794**
	Grim-PGS by Acceptance	–	–	1.014	0.933, 1.101
	Sex	**0.482**	**0.37, 0.628**	**0.492**	**0.379, 0.64**
	Income	**0.88**	**0.833, 0.93**	**0.875**	**0.828, 0.924**
	Pubertal Status	1.151	1.00 1.325	1.147	0.999, 1.318

Grim-PGS, polygenic score representing genetic risk for GrimAge; ALE, Adverse Life Events. Bolded estimates indicate statistical significance. Parallel models were conducted to examine the effect of adverse life events and parental acceptance separately.

**Table 4:** Associations between Grim-PGS, adverse life events, and depressive symptom trajectories among African American youth.

Comparison	Variable	OR	95% CI	OR	95% CI
High-Decreasing vs. Low	Grim-PGS	0.993	0.561, 1.759	1.122	0.717, 1.754
	Adverse life Events	1.202	0.859, 1.681	–	–
	Grim-PGS by ALE	1.322	0.904, 1.933	–	–
	Parent Acceptance	–	–	0.949	0.673, 1.34
	Grim-PGS by Acceptance	–	–	0.959	0.686, 1.339
	Sex	0.671	0.24, 1.879	0.707	0.251, 1.991
	Income	**0.834**	**0.712, 0.977**	**0.825**	**0.7, 0.973**
	Pubertal Status	1.38	0.923, 2.065	1.363	0.909, 2.046
Moderate vs. Low	Grim-PGS	0.952	0.782, 1.159	1.117	0.92, 1.356
	Adverse life Events	1.133	0.927, 1.385	–	–
	Grim-PGS by ALE	**1.377**	**1.159, 1.637**	**-**	**-**
	Parent Acceptance	–	–	0.873	0.737, 1.034
	Grim-PGS by Acceptance	–	–	1.086	0.952, 1.24
	Sex	0.881	0.527, 1.47	0.94	0.556, 1.588
	Income	0.979	0.897, 1.069	0.976	0.895, 1.065
	Pubertal Status	0.978	0.735, 1.30	0.988	0.746, 1.309

Grim-PGS, polygenic score representing genetic risk for GrimAge; ALE, Adverse Life Events. Bolded estimates indicate statistical significance. Parallel models were conducted to examine the effect of adverse life events and parental acceptance separately.

Within the AA subgroup (see **Table 4**), there were no significant main effects of adverse life events or parental acceptance on depression trajectories. However, similar to the finding among EA youth, the Grim-PGS by adverse life event interaction was also associated with greater likelihood of being in the moderate vs. low trajectory. Specifically, the association between Grim-PRS and membership in the moderate vs. low trajectory was stronger among youth who reported experiencing more adverse life events (+.5SD; OR = 1.44, 95% CI [1.05, 1.90]) than among youth who experienced fewer adverse life events (-.5SD; OR = 1.38, 95% CI [.27, 7.17]).

Among LX youth (see **Table 5**), parental acceptance was associated with lower likelihood of membership in the high trajectory of depressive symptoms relative to the low trajectory. No main effect of adverse life events nor GXE effects were detected.

**Table 5:** Associations between Grim-PGS, adverse life events, and depressive symptom trajectories among Latinx youth.

Comparison	Variable	OR	95% CI	OR	95% CI
High-Decreasing vs. Low	Grim-PGS	0.99	0.8, 1.225	0.994	0.786, 1.257
	Adverse life Events	1.06	0.88, 1.276	–	–
	Grim-PGS by ALE	1.006	0.829, 1.221	–	–
	Parent Acceptance	–	–	**0.816**	**0.671, 0.993**
	Grim-PGS by Acceptance	–	–	1.04	0.891, 1.215
	Sex	0.755	0.464, 1.228	0.76	0.468, 1.235
	Income	**0.878**	**0.807, 0.955**	**0.88**	**0.807, 0.96**
	Pubertal Status	1.224	0.96, 1.561	1.22	0.959, 1.552

Grim-PGS, polygenic score representing genetic risk for GrimAge; ALE, Adverse Life Events. Bolded estimates indicate statistical significance. Parallel models were conducted to examine the effect of adverse life events and parental acceptance separately.

## 4 Discussion

The goal of the current study was to examine whether environmental risk (i.e., adverse life events) and resilience factors (i.e., parental acceptance), moderate the influence of a genetic predisposition for epigenetic aging on trajectories of depression across early adolescence. In both the EA and AA subgroups we found adverse life events, but not parental acceptance, moderated polygenic scores for GrimAge such that there was increased likelihood of membership in a higher vs. lower depression trajectory for those high on both polygenic predisposition and adverse life events. This novel finding highlights how early life adversity may have long-term impacts on youth mental health. This finding is in-line with a Diathesis Stress Theory perspective, that adverse life events sometimes exacerbate genetic predispositions leading to psychopathology later in life. Findings have important implications for basic science and future research endeavors.

Using a growth mixture modelling approach, we identified heterogeneity in depressive symptom trajectories within EA, AA, and LX subgroups from age 10/11 to 12/13. The trajectories we identified largely replicate those found in our previous study in ABCD that included four waves of data and focused on substance use intent and perceived harm (55). In the EA subgroup we identified high-decreasing, increasing, moderate, and low trajectories. Three of these trajectories were also identified in the AA subgroup; high-decreasing, moderate, and low. In the LX subgroup we also identified high-decreasing and low trajectories. This supports previous work finding heterogeneity in depressive symptom trajectories and illustrates that there are important differences across race/ethnicity, such as an increasing group present only in the EA subgroup (55–58). It should be noted that differences in trajectories across racial/ethnic groups may be due to sample size and statistical power. Future studies should examine depressive symptom trajectories in large samples of racially and ethnically diverse youth to further elucidate developmental differences in youth’s depression.

Contrary to our hypothesis, we did not find direct effects of polygenic scores for GrimAge on depressive symptom trajectories in any subgroup. However, in zero-order correlations for the EA subgroup we did find a positive association between Grim-PGS and depressive symptoms at age 10/11. The lack of direct Grim-PGS associations with depressive symptoms may result from examining multiple trajectories of depressive symptoms, contributing to a loss in power. Alternatively, there are several intervening biological mechanisms between genetic predisposition for epigenetic aging and depression which may attenuate any direct association. Finally, as evidenced by our findings, it may be that genetic predisposition for GrimAge is only associated with depression when it is combined with exposure to adverse life events.

In the EA subgroup we found direct effects of adverse life events contributing to greater likelihood of depressive symptom trajectory membership in all trajectories compared to the low trajectory, which aligns with previous literature (15). As hypothesized, we found that adverse life events moderated the effect of polygenic predisposition for GrimAge contributing to greater likelihood of membership in the moderate vs. low depressive symptom trajectories in the EA and AA subgroups. This aligns with the Diathesis Stress Theory and past research indicating that adverse life events moderate genetic predispositions in predicting depressive symptoms and depression (18, 20). Recent reviews suggest that adverse life events may be associated with depression via genetic predispositions and epigenetic profiles embedded in serotonin, HPA axis, and oxytocin systems (19, 21, 59). Evidence also indicates that GrimAge is associated with brain aging and health, which could serve as one mechanism underlying effects on depression (4, 60, 61). Our findings extend prior work by examining polygenic predisposition for GrimAge epigenetic aging and by demonstrating associations with depressive symptom trajectories across early adolescence.

Of note, we did not find interactive effects with other trajectories with elevated depressive symptoms (e.g., high-decreasing, increasing). These trajectories may have other etiologic underpinnings. Alternatively, it may be that these subgroups were too small so we had limited power to detect interactive effects. Limited power may also explain the lack of any interaction effects in the LX subgroup. The lack of an ethnically aligned polygenic score also likely contributed to a lack of findings in this subgroup.

We did not find support for our hypothesis that parental acceptance, under Resilience Theory, would buffer the negative influence of polygenic predisposition for GrimAge on membership in higher vs. lower depressive symptom trajectories. It may be that the Diathesis Stress Theory fits better with depression and depressive symptoms, and parental acceptance has buffering effects on genetic predisposition for other child outcomes. However, in the EA and LX groups we did find direct effects of parental acceptance on higher likelihood of membership in the low depressive trajectory compared to higher depressive trajectories. These effects align with past research finding greater parental warmth and acceptance associated with lower childhood and adolescent depression (22–25). The lack of an interactive effect with parental acceptance may be due to the relatively large direct effects. Also, there is heterogeneity in the severity, timing, and type of adverse life events that youth experience. Accumulating research findings suggest that there may be sensitive periods in development during which different types or more severe adverse experiences can affect brain development, systemic inflammation, and adolescent depression (62–64). Therefore, the timing, severity, and type of adverse experiences may also affect epigenetic aging (65, 66).

In light of many strengths, including the large longitudinal sample, the focus on genetic influences on epigenetic aging, and the Bayesian methods that allowed us to form racially aligned polygenic scores, there were also several key weaknesses. First, as no GWAS on epigenetic aging exists in Latinx samples we were unable to create ethnically aligned polygenic scores for the LX group. Future genetically informed research should aim to include more ethnically diverse samples to ensure the equitable benefit of research findings. Second, modelling multiple trajectories in each racial/ethnic subgroup allowed use to examine for specificity of effects on distinct depressive symptom trajectories. However, this likely led to a loss of power in comparisons involving the smaller trajectories. Finally, we were only able to model trajectories using three waves of data because of the assessment timing of adverse life events. Future research examining effects on trajectories across developmental periods will help to illuminate distal effects of key risk and resilience factors.

## 5 Conclusion

We extended the literature by creating polygenic scores for GrimAge representing epigenetic aging. We found evidence of moderation by adverse life events in predicting membership in moderate vs. low depressive symptom trajectories across early adolescence. These findings highlight the detrimental role of adverse life events in exacerbating genetic risk for GrimAge epigenetic aging. Future research should identify developmental mechanisms and environmental contexts that facilitate epigenetic aging as a risk factor for depression across the lifespan. For instance, research could examine pathways from adverse life experiences, genetic-epigenetic effects, biological stress pathways, and depression across different developmental periods and in diverse sociocultural samples (67). Greater specificity in the mechanisms underlying this pathway can help inform prevention and intervention efforts. In the current study, findings underscore the need for continued preventive interventions for at-risk youth and support systems for youth exposed to adverse life events. In-line with the current findings, interventions that target individual and social mechanisms in youth exposed to adverse life events may buffer genetic influences on future mental health issues under a Diathesis-Stress Framework (68).

## Data Availability

Publicly available datasets were analyzed in this study. This data can be found here: National Institute for Mental Health Data Archive: https://nda.nih.gov/.
